# Understanding people's experiences of practising Tai Chi for health and wellbeing: a protocol for a qualitative systematic review

**DOI:** 10.3389/fmed.2026.1962809

**Published:** 2026-10-07

**Authors:** Hanjiang Liu, Weiting Liu, Youchang Zeng, Wurong Guo, Shun Chen, Lianyu Zou, Yanting Ding, Guoping Ye

**Affiliations:** 1 School of Qiaoxing Economics and Management, Fujian Polytechnic Normal University, Fuzhou, China; 2 School of Nursing, Peking University, Beijing, China; 3 School of Nursing and Midwifery, Edith Cowan University, Perth, WA, Australia; 4 Fujian Sports Vocational Education and Technical College, Fuzhou, China; 5 Reproductive Medicine Centre, Quanzhou Women’s and Children’s Hospital, Quanzhou, China; 6 Department of Traditional Chinese Medicine, Zhangzhou Affiliated Hospital of Fujian Medical University, Zhangzhou, China; 7 Department of Rehabilitation Medicine, Zhangzhou Affiliated Hospital of Fujian Medical University, Zhangzhou, China; 8 Department of Massage, Tai’an Hospital of Traditional Chinese Medicine, Tai’an, China; 9 Department of Acupuncture and Moxibustion, Xiamen TCM Hospital Affiliated to Fujian University of Traditional Chinese Medicine, Xiamen, China; 10 Department of Acupuncture and Moxibustion, Xiamen Hospital, Dongzhimen Hospital, Beijing University of Chinese Medicine, Xiamen, China; 11 Department of Acupuncture and Moxibustion, Xiamen Hospital of Traditional Chinese Medicine, Xiamen, China

**Keywords:** experience, health promotion, meta-aggregation, qualitative, Tai Chi

## Abstract

**Background:**

Tai Chi is a traditional Chinese mind-body practice that is widely used for health promotion, rehabilitation, symptom management, and overall wellbeing. Although numerous systematic reviews have demonstrated its potential effectiveness across a range of physical and psychological outcomes, quantitative evidence alone cannot explain how individuals experience Tai Chi, why they initiate or sustain practice, or how the practice is integrated into everyday life. Existing qualitative studies have provided valuable insights into participants' experiences; however, these findings remain dispersed across different populations, health conditions, and practice settings. To date, no qualitative evidence synthesis has comprehensively integrated people's experiences of practising Tai Chi across health-related contexts.

**Methods:**

This review will be conducted in accordance with the Joanna Briggs Institute (JBI) methodology for qualitative evidence synthesis using the meta-aggregation approach. A comprehensive search will be undertaken in MEDLINE, Embase, CINAHL, PsycINFO, AMED, Scopus, Web of Science, China National Knowledge Infrastructure (CNKI), Wanfang Data, Google Scholar, and ProQuest Dissertations & Theses Global from database inception to the final search date. Qualitative studies exploring people's experiences of practising Tai Chi for health and wellbeing will be eligible for inclusion. Two independent reviewers will undertake study selection, methodological quality appraisal, and data extraction. Methodological quality will be assessed using the JBI Critical Appraisal Checklist for Qualitative Research, and confidence in the synthesised findings will be evaluated using the ConQual approach.

**Discussion:**

This review will provide the first comprehensive qualitative evidence synthesis of people's experiences of practising Tai Chi across diverse populations and health-related contexts. The review is expected to generate evidence that informs the person-centred design, implementation, and future evaluation of Tai Chi programmes, while identifying priorities for future research.

**Systematic Review Registration:**

https://www.crd.york.ac.uk/PROSPERO/, PROSPERO CRD420261473258.

## 1 Introduction

Tai Chi is a traditional Chinese mind-body practice that integrates slow, intentional movements with postural alignment, regulated breathing, focused attention, and relaxation to promote the coordinated interaction of body and mind (1). Originating as a traditional Chinese martial art, Tai Chi has evolved into a widely practised mind-body exercise for health promotion, rehabilitation, symptom management, and general wellbeing (2). Its adaptability has enabled Tai Chi to be implemented across diverse populations and healthcare contexts, ranging from healthy community-dwelling adults to individuals living with chronic health conditions (3, 4). A substantial body of evidence has demonstrated benefits across a broad range of physical and psychological outcomes, including balance, functional capacity, pain, mood, sleep, quality of life, and fall prevention (4–6).

Despite the growing evidence base, little is known about why some individuals successfully integrate Tai Chi into everyday life whereas others discontinue practice despite experiencing clinical benefits. As a complex, multicomponent intervention, Tai Chi involves interacting physical, cognitive, behavioural, and social components that may influence how individuals engage with and experience the practice beyond its measurable clinical effects (1). Engagement with complex health interventions is influenced not only by their specific therapeutic components but also by the contextual factors surrounding care, including participants' expectations, previous experiences, therapeutic relationships, clinician communication, and the treatment environment, all of which may influence how interventions are experienced and translated into meaningful health outcomes (7, 8). Understanding how participants experience and interpret these contextual influences is therefore essential for developing and implementing person-centred Tai Chi programmes that align with individuals' needs, preferences, and lived experiences (9). Qualitative evidence syntheses of a non-pharmacological intervention further demonstrate that participants' experiences are dynamic rather than static, evolving throughout the intervention process as expectations, emotional responses, therapeutic relationships, and perceived benefits change over time (10). Such considerations suggest that understanding participants' experiences is essential to complement evidence of clinical effectiveness and optimise the person-centred implementation of Tai Chi programmes.

This complexity is reflected in the diversity of contexts in which Tai Chi is practised. It may be undertaken as part of a structured clinical or research programme, a supervised community activity, a home-based or digitally delivered programme, or a long-term personal health practice. Experiences may therefore differ according to health needs, prior exposure to Tai Chi, sociocultural background, practice environment, and stage of engagement. Existing qualitative studies provide empirical evidence of this complexity. Participants have described Tai Chi as supporting physical and psychological wellbeing, relaxation, body awareness, social connection, and a greater sense of mind-body integration (11, 12). At the same time, qualitative research has identified barriers to engagement and sustained practice, including the complexity of learning Tai Chi movements, instructor-related challenges, scheduling constraints, health-related limitations, safety concerns, and difficulties maintaining practice outside structured programmes (12, 13). Together, these findings indicate that participants' experiences are central to understanding the acceptability, implementation, and long-term sustainability of Tai Chi programmes and therefore warrant systematic qualitative synthesis.

Although individual qualitative studies have generated valuable insights, the evidence remains fragmented across populations, health conditions, and practice settings. Existing reviews of Tai Chi have largely focused on quantitative evidence concerning effectiveness, safety, and clinical or physiological outcomes, while participants' experiences have not been systematically integrated across health-related contexts. A pilot search of PROSPERO, PubMed, the Cochrane Database of Systematic Reviews, Joanna Briggs Institute (JBI) Evidence Synthesis, the Open Science Framework, and Google Scholar did not identify a published qualitative evidence synthesis addressing this question. Consequently, there remains a need to bring together qualitative evidence concerning how people experience, engage with, interpret, and sustain Tai Chi practice. By synthesising experiences across diverse populations and contexts, a qualitative evidence synthesis may provide a stronger evidence base to inform person-centred programme design, implementation, and future research.

Therefore, this qualitative systematic review aims to systematically identify, critically appraise, and synthesise qualitative evidence on people's experiences of practising Tai Chi for health and wellbeing. The review will address the following question: What are people's experiences of practising Tai Chi for health and wellbeing?

## 2 Methods

### 2.1 Study design

This qualitative systematic review will be conducted following the JBI methodology for qualitative evidence synthesis. This protocol is reported in accordance with the Preferred Reporting Items for Systematic Review and Meta-Analysis Protocols guidelines (14) and has been prospectively registered with PROSPERO (CRD420261473258).

### 2.2 Inclusion and exclusion criteria

Eligibility criteria will be structured according to the JBI PICo framework—Population, Phenomenon of Interest, and Context, with additional criteria specified for eligible study designs.

#### 2.2.1 Population

Studies involving individuals of any age who practise Tai Chi for health, wellbeing, rehabilitation, health promotion, or related purposes will be included.

For eligibility, a study will be considered health-related when improving, maintaining, or managing physical or psychological health or wellbeing is explicitly identified as a purpose or context of Tai Chi practice. Studies in which Tai Chi is practised for multiple purposes, such as both health and martial arts or cultural purposes, will be eligible where health or wellbeing constitutes an identifiable aspect of the practice or participants' reported experiences. Studies focusing on Tai Chi as a cultural practice, martial arts training, competitive activity, or educational practice without an identifiable health or wellbeing purpose will be excluded. No restrictions will be applied regarding age, sex, health status, or geographical location.

#### 2.2.2 Phenomenon of Interest

The phenomenon of interest is individuals' experiences of practising Tai Chi, including how they engage with, interpret, and sustain the practice. Eligible studies may explore participants’ perceptions, motivations, meanings, perceived benefits, challenges, and factors influencing continued participation.

#### 2.2.3 Context

Studies conducted in any setting and geographical location will be considered, including healthcare, rehabilitation, community, home-based, digital, and other settings in which Tai Chi is practised for health or wellbeing purposes.

#### 2.2.4 Types of studies

Qualitative studies employing recognised qualitative methodologies or designs, including phenomenology, grounded theory, ethnography, narrative inquiry, and qualitative descriptive research, will be included. Mixed-methods studies will be eligible when the qualitative component is reported separately and can be independently extracted.

### 2.3 Search strategy

The search strategy will aim to identify both published and unpublished studies. A three-step search strategy will be used in this review. First, pilot searches of MEDLINE (EBSCO), CINAHL (EBSCO) and China National Knowledge Infrastructure (CNKI) were undertaken in consultation with an academic librarian to identify relevant articles and refine the search terminology. The text words contained in the titles and abstracts of relevant articles, together with the index terms used to describe these articles, were used to develop the pilot search strategy. The search strategies developed for MEDLINE (EBSCO) and CNKI are presented in Supplementary Tables S1, 2, respectively.

Second, the identified concepts, keywords, and subject headings will subsequently be adapted for each database according to its specific controlled vocabulary, syntax, and language. Search strategies will combine controlled vocabulary (e.g., MeSH where applicable) with free-text keywords using Boolean operators (AND/OR), truncation, phrase searching, and other database-specific search techniques. The following electronic databases will be searched: MEDLINE (EBSCO), Embase, CINAHL (EBSCO), PsycINFO (EBSCO), Allied and Complementary Medicine Database (AMED), Scopus, Web of Science, CNKI, and Wanfang Data. Grey literature will be searched through Google Scholar and ProQuest Dissertations & Theses Global. No language restrictions will be applied. Databases will be searched from inception to the date of the final search.

Third, the reference lists of all included studies and relevant systematic reviews will be screened for additional eligible studies. Forward citation searching will also be conducted using Scopus, Web of Science, and Google Scholar. The first 200 Google Scholar records ranked by relevance will be screened for potentially eligible studies.

### 2.4 Study selection

All retrieved records will be uploaded directly into JBI System for the Unified Management, Assessment and Review of Information (JBI SUMARI, Adelaide, Australia) (15), where duplicates will be identified and removed. Titles and abstracts will be independently screened by two reviewers against the predefined eligibility criteria, and potentially eligible studies will subsequently undergo independent full-text assessment by two reviewers. Reasons for exclusion at the full-text stage will be documented. Disagreements will be resolved through discussion or consultation with a third reviewer. The study selection process will be reported in accordance with PRISMA 2020 and presented in a PRISMA flow diagram (16).

### 2.5 Assessment of methodological quality

Eligible qualitative studies and qualitative components of mixed-methods studies will be independently appraised by two reviewers using the JBI Critical Appraisal Checklist for Qualitative Research (17). Disagreements will be resolved through discussion or consultation with a third reviewer. Appraisal results will be reported in tabular and narrative form. In accordance with JBI methodology, methodological quality will not be used as a criterion for study exclusion.

### 2.6 Data extraction

Data will be independently extracted by two reviewers using the standardised JBI qualitative data extraction tool in JBI SUMARI. Extracted information will include study and participant characteristics, geographical and cultural context, characteristics of Tai Chi practice, qualitative methodology and methods, and findings relevant to the review question.

Findings and their supporting illustrations will be extracted verbatim and assigned a level of credibility based on the congruence between each finding and its supporting illustration. Findings will be classified as unequivocal when clearly supported by the illustration, credible when the relationship is plausible but open to challenge, or not supported when the finding is not supported by the illustration, in accordance with JBI guidance (18). Disagreements will be resolved through discussion or consultation with a third reviewer. Where necessary, corresponding authors will be contacted to clarify missing or unclear information.

### 2.7 Data synthesis

Qualitative findings will, where possible, be synthesised using the JBI meta-aggregation approach within JBI SUMARI (18). Findings and their supporting illustrations will be assembled and grouped into categories based on similarity in meaning. These categories will then be aggregated to generate synthesised findings that address the review question and may inform health practice, programme development, and future research. Differences in participant and practice characteristics, such as age, health condition, Tai Chi style, duration and mode of practice, and cultural context, will be considered during category formation and synthesis. Where sufficient studies and data are available, similarities and differences in experiences across populations and practice contexts will be explored and reflected in the categories and synthesised findings, as appropriate. Only findings rated as unequivocal or credible will be included in the synthesis. Where findings cannot be meaningfully aggregated, they will be presented narratively.

### 2.8 Assessing confidence in the findings

Confidence in the synthesised findings will be assessed using the JBI ConQual approach (19). The overall confidence in each synthesised finding will be determined based on dependability and credibility, and the results will be presented in a ConQual Summary of Findings table.

### 2.9 Management of non-english studies

No studies will be excluded on the basis of language. Studies from CNKI and Wanfang databases will be screened, critically appraised, and extracted from the original-language reports by bilingual reviewers proficient in both Chinese and English. Relevant qualitative findings and participant quotations will be translated into English for synthesis, with translations checked against the original Chinese text by a second bilingual reviewer to ensure accuracy and minimise potential loss or alteration of meaning. For studies published in languages other than English or Chinese, appropriate translation support will be sought as required for study selection, critical appraisal, and data extraction.

### 2.10 Reflexivity

Reflexivity will be considered before and throughout the review process. The review team will critically reflect on how their professional and cultural backgrounds, prior experiences, and assumptions may influence the review process, particularly data extraction, categorisation, and synthesis. Regular team discussions and reflexive notes will be used to document and reflect on key methodological and analytical decisions.

## 3 Ethics and dissemination

Ethical approval is not required because this review will synthesise data from previously published studies and will not involve the collection of primary data from human participants. The review will be conducted in accordance with the JBI methodology for qualitative evidence synthesis and reported in accordance with the PRISMA 2020 statement (16). The findings will be disseminated through publication in a peer-reviewed journal and presentations at national and international scientific conferences.

## 4 Discussion

Despite growing evidence supporting the effectiveness of Tai Chi, understanding how individuals experience and interpret the practice remains comparatively underdeveloped. The purpose of this review is therefore not to re-evaluate effectiveness, but to address the complementary question of how Tai Chi is experienced across diverse populations and real-world contexts. Qualitative evidence synthesis can integrate participants' perspectives and contextual experiences that may not be captured through quantitative outcomes alone (10).

The proposed review will follow the JBI methodology for qualitative evidence synthesis using the meta-aggregation approach. This approach is particularly appropriate because it aims to preserve participants' original meanings while generating synthesised findings that are directly applicable to health practice and decision-making. Rather than generating new theoretical interpretations, the review will aggregate qualitative findings across studies to produce synthesised statements representing participants' experiences of practising Tai Chi. Assessing methodological quality using the JBI Critical Appraisal Checklist and confidence in the synthesised findings using the ConQual approach will further enhance the transparency, credibility, and applicability of the review findings.

Beyond informing practice, this review is expected to strengthen the qualitative evidence base on Tai Chi by identifying common experiential patterns and context-specific perspectives across different populations, health conditions, and practice settings. The findings may also inform researchers, clinicians, Tai Chi instructors, and programme developers in designing and implementing more person-centred Tai Chi programmes that are responsive to participants' needs, preferences, and experiences, while highlighting priorities for future qualitative research.

## 5 Strengths and limitations

This protocol has several strengths. To our knowledge, it will be the first qualitative systematic review to comprehensively synthesise people's experiences of practising Tai Chi across diverse populations, health conditions, and practice settings. The review will be conducted using the JBI methodology for qualitative evidence synthesis, with methodological quality appraised using the JBI Critical Appraisal Checklist and confidence in the synthesised findings assessed using the ConQual approach. These methods are expected to enhance the transparency, rigour, and trustworthiness of the review process and findings.

Several limitations should also be acknowledged. The review will depend on the methodological quality and reporting of the included primary studies, which may affect the credibility and richness of the available qualitative evidence. Variations in participant characteristics, health conditions, Tai Chi styles, intervention characteristics, and sociocultural contexts, which may be amplified by the broad eligibility criteria, may limit direct comparisons and the transferability of individual findings. Despite procedures to support translation accuracy, some nuances of meaning may be lost when qualitative findings are translated across languages and cultural contexts. Finally, screening only the first 200 Google Scholar records ranked by relevance may result in some relevant grey literature being missed.
