# Supplementary material for: Understanding people's experiences of practising Tai Chi for health and wellbeing: a protocol for a qualitative systematic review
Source: Front Med (Lausanne). 2026 Oct 7;13:1962809. doi: 10.3389/fmed.2026.1962809 (PMC13683576; doi:10.3389/fmed.2026.1962809)
Supplement: Supplementary file 1 [file Table1.docx]

Supplementary Table 1. Search strategy developed for MEDLINE (EBSCO)

| **Search number** | **Term searched** | **Number of hits** |
| --- | --- | --- |
| S1 | MH “Tai Ji” | 1,835 |
| S2 | XB “Tai Ji” | 84 |
| S3 | XB “Tai Ji Quan” | 52 |
| S4 | XB Taijiquan | 68 |
| S5 | XB “Tai Chi” | 3,097 |
| S6 | XB “Tai Chi Chuan” | 256 |
| S7 | XB “Tai Chi Quan” | 14 |
| S8 | XB Taichi | 116 |
| S9 | S1 OR S2 OR S3 OR S4 OR S5 OR S6 OR S7 OR S8 | 3,520 |
| S10 | XB Experience* | 1,699,667 |
| S11 | XB “Lived experience*” | 24,467 |
| S12 | XB Perspective* | 614,935 |
| S13 | XB Perception* | 415,564 |
| S14 | XB View* | 651,492 |
| S15 | XB Belief* | 129,349 |
| S16 | XB Opinion* | 139,010 |
| S17 | XB Attitude* | 232,440 |
| S18 | XB Meaning* | 236,810 |
| S19 | XB Satisfaction* | 237,922 |
| S20 | XB Acceptability | 73,156 |
| S21 | XB Engagement* | 169,900 |
| S22 | XB Adherence | 226,943 |
| S23 | XB Qualitative | 434,649 |
| S24 | XB Interview* | 568,331 |
| S25 | XB “Focus group*” | 93,989 |
| S26 | XB Barrier* | 568,076 |
| S27 | XB Facilitator* | 56,981 |
| S28 | XB Motivation* | 129,718 |
| S29 | S10 OR S11 OR S12 OR S13 OR S14 OR S15 OR S16 OR S17 OR S18 OR S19 OR S20 OR S21 OR S22 OR S23 OR S24 OR S25 OR S26 OR S27 OR S28 | 4,826,001 |
| S30 | S9 AND S29 | 891 |

*Note*. Pilot search undertaken on 5 September 2026, from database inception to the search date. In MEDLINE (EBSCO), “XB” indicates a search of the title or abstract fields.

Supplementary Table 2. Search strategy for China National Knowledge Infrastructure (CNKI)

| **Search number** | **Term searched** | **Number of hits** |
| --- | --- | --- |
| S1 | Subject: 太极 + 太极拳 | 36,660 |
| S2 | Subject: 体验 + 经历 + 感受 + 观点 + 看法 + 态度 + 信念 + 动机 + 接受度 + 依从性 + 定性 + 质性 + 访谈 + 焦点小组 | 2,363,213 |
| S3 | S1 AND S2 | 1,833 |

*Note*. Pilot search undertaken on 5 September 2026, from database inception to the search date. Search field: Subject; search mode: Exact; search scope: All databases. In CNKI advanced search interface, “+” represents the Boolean operator OR.
